# Neoadjuvant concurrent chemoradiotherapy followed by transanal total mesorectal excision assisted by single-port laparoscopic surgery for low-lying rectal adenocarcinoma: a single center study

**DOI:** 10.1186/s12957-020-01980-y

**Published:** 2020-08-11

**Authors:** Yen-Jung Lu, Chien-Hsin Chen, En-Kwang Lin, Szu-Yuan Wu

**Affiliations:** 1 Department of Colorectal Surgery, Wan Fang Hospital, Taipei Medical University, Taipei, Taiwan; 2 Department of Food Nutrition and Health Biotechnology, College of Medical and Health Science, Asia University, Taichung, Taiwan; 3 Division of Radiation Oncology, Lo-Hsu Medical Foundation, Lotung Poh-Ai Hospital, No. 83, Nanchang St., Luodong Township, Yilan County, 265 Taiwan; 4 Big Data Center, Lo-Hsu Medical Foundation, Lotung Poh-Ai Hospital, Yilan, Taiwan; 5 Department of Healthcare Administration, College of Medical and Health Science, Asia University, Taichung, Taiwan; 6 Cancer Center, Lo-Hsu Medical Foundation, Lotung Poh-Ai Hospital, Yilan, Taiwan; 7 School of Dentistry, College of Oral Medicine, Taipei Medical University, Taipei, Taiwan

**Keywords:** Neoadjuvant CCRT, TaTME-SPLS, Rectal adenocarcinoma

## Abstract

**Purpose:**

To assess the feasibility and short-term outcomes of neoadjuvant chemoradiotherapy (CCRT) followed by transanal total mesorectal excision assisted by single-port laparoscopic surgery (TaTME-SPLS) for low-lying rectal adenocarcinoma.

**Methods and materials:**

A total of 23 patients with clinical stage II-III low-lying (from anal verge 0-8 cm) rectal adenocarcinoma who underwent neoadjuvant CCRT followed by TaTME-SPLS consecutively from December 2015 to December 2018, were enrolled into our study. Chi-squared testing and Student’s *t* testing were used to make parametric comparisons, and Fisher’s exact test or the Mann–Whitney *U* test were used to make nonparametric comparisons.

**Results:**

Conversion rate in patients who underwent neoadjuvant CCRT followed by TaTME-SPLS was only 4%. The mean operation time was 366 min and the inter-sphincter resection (ISR) was done for 14 patients (60%). The mean number of lymph nodes harvested was 15. There was no surgical mortality, but the 30-day morbidity rate was 21% (5 patients were Clavien-Dindo I-II). Pathological complete response was 21.74% with 100% organ preservation and 100% clear distal margin after neoadjuvant CCRT followed by TaTME-SPLS.

**Conclusion:**

TaTME-SPLS would be highly successful in lymph node negative and low T stage of low-lying rectal cancer patients who had pathological complete remission or high percentage of partial remission after neoadjuvant CCRT.

## Introduction

Laparoscopic colon resection has been considered as an alternative procedure to open colon resection after a series of randomized controlled trials (RCTs) from 2004 to 2005, with short-term advantages, like less morbidity and hospital stay, but similar long-term survival [1–3]. However, there have been controversial conclusions regarding laparoscopic total mesorectal excision (TME) and open TME in patients with rectal cancer [1–7]. With progression in contemporary surgical techniques and equipment, laparoscopic TME was found to be safe and equivalent in terms of long-term outcomes, compared with open TME in 2 RCTs from 2014 to 2015 [4, 5]. However, other RCTs failed to present the advantages of laparoscopic TME [6, 7]. Therefore, laparoscopic TME has still been a surgical challenge for middle and low rectal cancer, which requires surgeons experienced with high-volume of cases for treatment of selective patients [8]. The conversion rate of laparoscopic TME was up to 17%, and an abdomino-perineal combined resection (APR) rate was up to 29% in 2015 trial [4]. Previous studies still have some unsolved problems regarding laparoscopic TME [4–7].

Transanal total mesorectal excision (TaTME) was a new surgical procedure first introduced in 2010 [9], which has received much attention recently. TaTME provides a better view with “down-to-up” mesorectal dissection via transanal single-port platform [10, 11]. TaTME can confirm the tumor location, achieve enough distal margin, and better specimen quality [12–14]. TaTME has improved visualization of the pillars, plexuses, and neurovascular bundles that may secure these structures and sexual function far better [15, 16]. Therefore, Heald in 2013 revealed that TaTME may be a new solution to preserve organ and achieve superior clear margin and better sexual function in middle or lower third rectal cancer treatment [17]. When TaTME was performed via the transanal platform, it was difficult to continue upward dissection to reach the descending colon or splenic flexure [17, 18]. So, the procedures of TaTME, including high ligation of the inferior mesenteric artery and vein and mobilization of the sigmoid colon, descending colon, and splenic flexure colon were performed assisted by the traditional laparoscopic surgery or single-port laparoscopic surgery [17, 18]. According to our knowledge, there were few case series reports on TaTME assisted by single-port laparoscopic surgery (TaTME-SPLS) [17, 18].

In the current study, we investigated the feasibility and short-term clinical outcomes of neoadjuvant CCRT followed by TaTME-SPLS in Asian patients with middle to lower third rectal adenocarcinoma. We also estimated the differences of TaTME-SPLS in Western and Eastern patients with middle to lower third rectal adenocarcinoma.

## Patients and methods

### Patients

According to our previous studies, patients were proven to have rectal adenocarcinoma histologically if they had the middle to lower third tumor margin within 8 cm from anal verge pre-operatively as measured by rigid sigmoidoscopy; and those diagnosed with rectal adenocarcinoma between December 2015 and December 2018, at Taipei Medical University-Wan Fang Hospital were included in this study [19–22]. The inclusion criteria included patients with clinical stage II to III cancer who underwent neoadjuvant CCRT followed by TaTME-SPLS. All rectal cancer staging protocols included performing a magnetic resonance imaging scan prior to surgery to confirm the tumor necrosis and metastasis staging of rectal adenocarcinoma. The exclusion criteria included patients who had only local excision, laparoscopic or robotic TME, TaTME assisted by multiple port laparoscopic surgery, and APR. At our hospital, neoadjuvant CCRT is indicated and is standard for all patients with clinical stage II or III of middle to lower third rectal cancer. Standard and consistent neoadjuvant CCRT consists of a cumulative radiation dosage of 50.40 Gy in 28 fractions and a dosage of 1000 mg fluorouracil per square meter per day during the first and fifth weeks of radiotherapy [23]. All RT technique was done with intensity-modulated radiation therapy.

Surgery was usually performed 6-10 weeks after the completion of neoadjuvant CCRT. Patients were given information regarding the expected benefits and potential risks of the procedures and an informed consent was obtained from all participants included in the study. Approval for this study was obtained from the Institutional Review Board of Taipei University-Wan Fang Hospital (JIRB N201906057).

### Surgical technique

#### Trans-anal endoscopic dissection

The patient was placed in the lithotomy position. A Lone Star Retractor® (Cooper Surgical, Trumbull, CT, USA) was placed to expose the anal canal. A transanal single-port device, the Gelpoint path® (Applied Medical, Rancho, Santa Margarita, CA, USA), was positioned. Three trocars were inserted via the Gelpoint path®, and a flexible endoscope was inserted via a trocar. Under endoscopic guidance, the tumor was identified and the rectum was closed circumferentially at least 10 mm distal to the lower margin of the tumor by purse-string suture to avoid tumor spillage. Then, the rectum was divided circumferentially.

If a tumor is located near the dentate line of the anus, an inter-sphincter resection (ISR) is necessary [24]. Conventional dissection was started at the level of inter-sphincter groove circumferentially, to ensure at least 10 mm distal safe margin. Then, subsequently, the rectal lumen was closed with a purse-string suture. The Gelpoint path® was placed into the anal canal. The peri-mesorectal space was insufflated with CO_2_ with a low flow (1.5 L/min) and a maximal pressure of 12 mmHg. According to the principle of TME, “bottom-to-up” dissection was performed under endoscopic guidance to achieve the rectum mobilization circumferentially [25]. Then, the peritoneum of Douglas’ pouch was opened to enter into the peritoneal cavity via dissection.

#### Trans-abdominal dissection

All the patients were placed in the lithotomy position. A 4-cm transverse incision was performed at the right lower quadrant of the abdomen, the site of the future ileostomy. The wound was deepened to enter the peritoneal cavity and an abdominal Gelpoint system® (Applied Medical, Rancho, Santa Margarita, CA, USA), was inserted into the peritoneal cavity. A flexible laparoscope, 10 mm in diameter, and only standard straight laparoscopic instruments were used. The abdomen was insufflated with CO_2_ to a pressure of 12 mmHg. The operation table was rotated toward the right side. The sigmoid colon and rectum were mobilized using the medial to lateral approach, and the inferior mesenteric pedicle isolation was performed. The roots of the inferior mesenteric artery and vein were ligated with laparoscopic clips, and then, were divided. Extensive mobilization of the splenic flexure and transverse colon was performed in selective patients. For this step, an additional 5-mm trocar was inserted at the site of the future pelvic drain in the left iliac fossa. Finally, the total mobilization of the rectum was performed to meet the plane created by transanal dissection, and the specimen was completely freed. Extraction of all specimens was performed through the abdominal single-port. The sigmoid colon was clamped and divided. TaTME was performed. In selective patients, a transverse coloplasty pouch (TCP) was created for colon-anal anastomosis using the sigmoid colon [26]. The rectal reconstruction was performed by the colon-anal hand-sewn anastomosis or the colon-rectal anastomosis with the transanal insertion of a circular stapler. Usually, a pelvic drain was placed. The loop ileostomy was performed at the site of the insertion of the single port abdominal device.

### Statistical analysis

Frequency tables were used for patients’ presentations and tumor characteristics. We used the two-tailed chi-square test for differences in proportions and the Student’s *t* test for continuous numerical variables. Statistical significance was defined as a value of *P* < 0.05. We compared all study data with Statistical Package for the Social Sciences (SPSS) version 13.0 for Windows (SPSS Inc., Chicago, IL, USA).

## Results

A total of 23 patients underwent neoadjuvant CCRT followed by TaTME-SPLS, and one patient underwent open TME. The demographic and tumor characteristics are presented in Table 1. In our study, patient characteristics were as follows: 56.52% were male patients, 82.61% had ASA score 2, the median distance from the anal verge was 52 mm, the median tumor size was 20 mm, 65.22% were at AJCC clinical stage III, 91.30% were at cT3, and 47.83% were at cN1; the characteristics of our patients were compatible with only one TaTME-SPLS study done in France [18]. Sixty percent of the patients had ISR because their tumor margins were close to the dentate line of the anus. The mean operation time was 366 min. No intra-operative complications occurred in our study. Five patients experienced complications (21.74%), and they were all minor complications according to the classification of Clavien-Dindo: 3 patients had urinary tract infection, one patient had ileus, and one patient had a pelvic abscess [27]. No patient needed another surgical or radiological intervention to treat the complications. No 30-day mortality occurred in our study. The mean hospital stay was 8.3 days (Table 1). The median follow-up time is 13.45 months and all follow-up time in these 23 patients was over 6 months. All RT technique was done with intensity-modulated radiation therapy. Twenty-three patients could tolerate the whole courses of CCRT and the median total cumulative dose of fluorouracil were 5000 mg per square meter. Moreover, the standard RT dose 50.4Gy could be completed in all 23 patients.

**Table 1:** Characteristics of patients with middle to lower third rectal adenocarcinoma, receiving neoadjuvant concurrent chemoradiotherapy followed by trans-anal total mesorectal excision assisted by single port laparoscopic surgery

	*n* (%)
Sex	
Male	13 (56.52)
Female	10 (43.48)
Age, median (range)	63 (31-80)
BMI (kg/m^2^), median (range)	23.7 (18-32)
ASA	
ASA score 1	1 (4.35)
ASA score 2	19 (82.61)
ASA score 3	3 (13.04)
Distance to anal verge (mm), median (range)	54 (20-80)
Tumor size (mm), median (range)	20 (6-85)
AJCC clinical stages	
Stage II	8 (34.78)
Stage III	15 (65.22)
Clinical T stage	
cT2	2 (8.70)
cT3	21 (91.30)
Clinical N stage	
cN0	8 (34.78)
cN1	11 (47.83)
cN2	4 (17.39)
Neoadjuvant CCRT	23 (100.00)

*ASA* American Society of Anesthesiologists, *BMI* body mass index, *CCRT* concurrent chemoradiotherapy, *AJCC* the American Joint Committee on Cancer

The pathological findings after neoadjuvant CCRT followed by TaTME-SPLS are presented in Table 2. In this study, the pathologic complete response was 21.74%. R0 resections were achieved in 23 patients (100%). Circumferential resection margin (CRM) was positive in 2 patients (8.70%). There were only two patients with clinical T3 stages having R1 resection in the circumferential margin, and all 23 patients were R0 resection in the distal margin. The margin status (R0 or R1 resection) might be associated with the initial T stage in our study, because the only two patients with R1 resection margin were initial clinical T3 stage. No incomplete or perforated specimen was present in this series. In our study, 82.61% had mobilization of the splenic flexure, 60.87% had ISR, 39.13% had stapled type of anastomosis, the mean number of lymph nodes harvested was 15, and the mean volume of blood loss was 159 mL in patients with middle to lower third rectal adenocarcinoma who underwent neoadjuvant CCRT followed by TaTME-SPLS.

**Table 2:** Clinical outcomes of patients with middle to lower third rectal adenocarcinoma receiving neoadjuvant concurrent chemoradiotherapy followed by trans-anal total mesorectal excision assisted by single port laparoscopic surgery

	***n*** (%)
Pathologic T stages	
ypT0	5 (21.74)
ypT1	5 (21.74)
ypT2	9 (39.13)
ypT3	4 (17.39)
Pathologic N stages	
ypN0	19 (82.61)
ypN1	4 (17.39)
Pathologic response	
Complete response	5 (21.74)
Partial response	18 (78.26)
No response	0 (0.00)
Distal margin status	
R0	23 (100.00)
R1	0 (0.00)
R2	0 (0.00)
Circumferential resection margin status	
R0	21 (91.30)
R1	2 (8.70)
R2	0 (0.00)
Lymph nodes harvested, mean (range)	15.3 (6-42)
Operative time (min), mean (range)	366 (240-480)
Estimated blood loss (mL), mean (range)	159 (10-650)
Conversion	1 (4.35)
30-days complication	5 (21.74)
Mobilization of splenic flexure	19 (82.61)
Inter-sphincter resection	14 (60.87)
Type of anastomosis (hand-sewn)	14 (60.87)
Type of anastomosis (stapled)	9 (39.13)
Length of hospital stays (days), mean (range)	8.4 (6-22)

Comparing with the 2 leading studies based on Western and Eastern country patients with middle to lower third rectal adenocarcinoma, receiving TaTME-SPLS, higher rates of neoadjuvant CCRT, mobilization of the splenic flexure, ISR, and stapled types of anastomosis were found in our study (Table 3). In our study, downstaging effects of T and N stage, better pathologic response, and R0 resection margin significantly increased in all patients after receiving neoadjuvant CCRT (Table 3).

**Table 3:** Comparison of the leading two series study of patients in Western and Eastern countries with middle to lower third rectal adenocarcinoma, receiving trans-anal total mesorectal excision assisted by single port laparoscopic surgery

	Meillat et al.	The present study	*P* value
Follow-up intervals	1/2012–4/2015	12/2015-12/2018	
Case numbers	41	23	
Age	64	63	N/A
Sex			0.459***
Male	27 (65.85%)	13 (56.52%)	
Female	14 (34.15%)	10 (43.48%)	
BMI	24	23	N/A
Neoadjuvant CCRT	30/41 (73.17%)	23/23 (100.00%)	0.005**
Operative time (min)	358	366	N/A
Location of single port			
Ileostomy site	100%	100%	1.000**
Mobilization of splenic flexure	6/41 (14.63%)	19/23 (82.61%)	< 0.001**
Conversion	1/41 (2.44%)	1/23 (4.35%)	0.851*
Complication	10/41 (24.39%)	5/23 (21.74%)	0.815*
Type of anastomosis			< 0.001***
Hand-sewn	41/41 (100.00%)	14/23 (60.87%)	
Stapled	0 (0.00%)	9/23 (39.13%)	
Lymph nodes harvested	13	15	N/A
Pathologic T stages			< 0.001***
ypT0	11 (26.83%)	5 (21.74%)	
ypT1	4 (9.76%)	5 (21.74%)	
ypT2	11 (26.83%)	9 (39.13%)	
ypT3	15 (36.59%)	4 (17.39%)	
Pathologic N stages			< 0.001***
ypN0	10 (23.39%)	19 (82.61%)	
ypN1	31 (75.61%)	4 (17.39%)	
Distal margin status			< 0.001*
R0	38 (92.68%)	23 (100.00)	
R1	3 (7.32%)	0 (0.00)	
Inter-sphincteric resection	15/41 (36.59%)	14/23 (60.87%)	< 0.044**
Length of hospital stays (days)	10	8.4	N/A

*N/A* not available, *BMI* body mass index, *CCRT* concurrent chemoradiotherapy

*Fisher’s exact test

**Student’s *t* test

***Chi-squared test

## Discussion

In 2011, Tuech et al. described the first case of TaTME-SPLS in the world [28]. Then, Dumont et al. presented 2 case reports in 2012 [29]. Later on, TaTME-SPLS case reports were presented by 2 teams in 2013 and 2015 [30, 31]. Until 2017, Meillat et al. had published the first retrospective study of 41 patients who underwent TaTME-SPLS, rather than case reports [18]. Our study will be the second retrospective study for TaTME-SPLS in the world and the first retrospective study of neoadjuvant CCRT followed by TaTME-SPLS in Asian patients with middle to lower third rectal adenocarcinoma (within 8 cm from anal verge measured by rigid sigmoidoscopy based on our previous studies) [19–22]. Meillat et al. revealed a low conversion rate (2%), high sphincter preservation rate (100%), and an acceptable 30-day morbidity rate (24%) in 41 patients [18]. In comparison with the previous study, our study had similar results for age, BMI, tumor location, operative time, location of ileostomy, conversion rate, 30-day complication rate, 30-day mortality rate, number of lymph nodes harvested, and length of hospital stay (Table 3). However, we had a higher rate of neoadjuvant CCRT (100%), mobilization of the splenic flexure (65.22%), ISR (60.87%), stapled types of anastomosis (39.13%), and better pathologic response with downstaging in our study (Table 3).

After the first single-port laparoscopic right hemi-colectomy had been described in 2008 [32], several studies suggested the benefits of single-port laparoscopic colectomy over multiple port laparoscopic surgery, which included better cosmetic outcomes, less postoperative pain, and faster postoperative recovery [33, 34]. Otherwise, reports that described TME-SPLS for rectal cancer were limited. A report by Tei et al. in 2015 discussed short-term outcomes of TME-SPLS in 50 patients with rectal cancer [35]. Tei et al. concluded that TME-SPLS is a safe and feasible procedure for selective patients with rectal cancer. The middle to lower third rectal cancer was excluded because of technical difficulty [35]. At present, we might suggest that neoadjuvant CCRT followed by TaTME-SPLS may be the solution to TME-SPLS for low-lying rectal cancer with 100% organ preservation, 100% clean distal margin, and superior pathologic response.

LAR involves removal of the sigmoid colon and rectum to a level, where the distal margin is free of cancer, followed by a primary anastomosis between the descending colon and the rectum (colorectal anastomosis) or the anal sphincter (colo-anal anastomosis) [36, 37]. The splenic flexure must be mobilized for the descending colon to reach the deep pelvis for the anastomosis [36, 37]. Previous studies indicated that mobilization of the splenic flexure was mandatory, to ensure an optimal blood supply to the residual colon and a tension-free anastomosis [38, 39]. In effect, mobilization of the splenic flexure was mainly performed in patients with a short left mesentery [40]. However, mobilization of the splenic flexure was not free from intra-operative complications, like the spleen injury, and it increased the complexity of an already demanding operation, such as rectal resection [41–43]. In addition, limitations to TaTME included the inability to completely visualize the intracoelomic cavity, transect the inferior mesenteric artery in an oncologic high ligation fashion, and mobilization of the splenic flexure [15]. Most experts have recommended it for female patients with benign disease without prior radiotherapy [44–46]. However, in our study, neoadjuvant CCRT followed by TaTME-SPLS was performed smoothly in all patients with middle to lower third rectal adenocarcinoma (from anal verge 0-8 cm measured by rigid sigmoidoscopy), including 82.61% patients, in whom mobilization of the splenic flexure was performed. Our findings imply that neoadjuvant CCRT did not increase difficulty and comorbidities in patients with low-lying rectal adenocarcinoma, receiving TaTME-SPLS (Table 3).

Patients with low-lying rectal adenocarcinoma (from anal verge 0-8 cm measured by rigid sigmoidoscopy), receiving TaTME-SPLS had better pathologic complete response rate and more downstaging of pathologic T and N stages, compared with the Meillat et al. study (Tables 2 and 3). In our study, neoadjuvant CCRT might be contributing to better pathologic response and clean distal margin in all patients, compared with the Meillat et al. study [18]. The pathologic complete response rate (21.74%) after neoadjuvant CCRT in this study was compatible with the previous studies [47–49]. According to previous studies, better pathologic complete response rate after neoadjuvant CCRT was associated with superior survival outcomes [47, 48, 50–52]. Our outcomes of neoadjuvant CCRT followed by TaTME-SPLS in patients with low-lying rectal cancer can be promising and might be compatible with previous studies; we need longer follow-up time to verify the better survival in our study [51, 52]. Our findings suggest that neoadjuvant CCRT followed by TaTME-SPLS in patients with low-lying rectal adenocarcinoma might bring in better organ preservation, clean distal margin, tolerable toxicities, and superior pathologic complete response with potential better overall survival [51, 52].

This study had some strengths. Prior to this study, no clinical data had proven that neoadjuvant CCRT followed by TaTME-SPLS in patients with low-lying rectal adenocarcinoma leads to excellent organ preservation, clean distal margin, acceptable toxicities, and good pathologic complete response; this study provides novel data. Our definition of low-lying rectal adenocarcinoma was clear (from anal verge 0-8 cm), and measuring tools were consistent, that is, rigid sigmoidoscopy. Our regiments of neoadjuvant CCRT, the interval from completion of CCRT to surgery, and procedures of TaTME-SPLS were consistent and without discrepancy treatments. Our study was the first study to evaluate the effect of neoadjuvant CCRT followed by TaTME-SPLS in patients with low-lying rectal adenocarcinoma. Additionally, this is also the first study that presents neoadjuvant CCRT followed by TaTME-SPLS as safe and effective for organ preservation without inadequate margin in Asian patients with low-lying rectal adenocarcinoma.

This study had some limitations. First, in this study, the sample size of patients with low-lying rectal adenocarcinoma who underwent neoadjuvant CCRT followed by TaTME-SPLS was small. Second, all patients with low-lying rectal adenocarcinoma were enrolled from an Asian population, and so, the corresponding ethnic susceptibility remains unclear. Therefore, our results must be cautiously extrapolated to non-Asian populations. Third, surgeons not familiar with this technique needed standardized training, including observations, cadaveric labs or hands-on courses, and proctorship or mentorship, with early case experiences [44, 45]. Patient-volume for surgeons familiar with neoadjuvant CCRT followed by TaTME-SPLS might be concerned.

## Conclusions

TaTME-SPLS would be highly successful in lymph node negative and low T stage of low-lying rectal cancer patients who had pathological complete remission or high percentage of partial remission after neoadjuvant CCRT.

## Data Availability

The data sets supporting the conclusions of this study are included within this article and supporting files.

## Abbreviations

CCRT: Chemoradiotherapy
TaTME-SPLS: Transanal total mesorectal excision assisted by single-port laparoscopic surgery
RCTs: Randomized controlled trials
TME: Total mesorectal excision
APR: Abdomino-perineal combined resection
TaTME: Transanal total mesorectal excision
TCP: Transverse coloplasty pouch
SPSS: Statistical Package for the Social Sciences
AJCC: The American Joint Committee on Cancer
CRM: Circumferential resection margin
ISR: Inter-sphincter resection
BMI: Body mass index
LAR: Low anterior resection
ASA: American Society of Anesthesiologists
